# Combinatorial Network of Transcriptional and miRNA Regulation in Colorectal Cancer

**DOI:** 10.3390/ijms24065356

**Published:** 2023-03-10

**Authors:** Rupesh Kumar, Maged Mostafa Mahmoud, Hanaa M. Tashkandi, Shafiul Haque, Steve Harakeh, Kalaiarasan Ponnusamy, Shazia Haider

**Affiliations:** 1 Department of Biotechnology, Jaypee Institute of Information Technology, A-10, Sector 62, Noida 201309, India; 20401005@mail.jiit.ac.in; 2 King Fahd Medical Research Center, King Abdulaziz University, Jeddah 21589, Saudi Arabia; 3 Department of Medical Laboratory Sciences, Faculty of Applied Medical Sciences, King Abdulaziz University, Jeddah 21589, Saudi Arabia; 4 Molecular Genetics and Enzymology Department, Human Genetics and Genome Research Institute, National Research Centre, Cairo 12622, Egypt; 5 Department of General Surgery, Faculty of Medicine, King Abdulaziz University, Jeddah 21589, Saudi Arabia; 6 Research and Scientific Studies Unit, College of Nursing and Allied Health Sciences, Jazan University, Jazan 45142, Saudi Arabia; 7 Gilbert and Rose-Marie Chagoury School of Medicine, Lebanese American University, Beirut 13-5053, Lebanon; 8 Centre of Medical and Bio-Allied Health Sciences Research, Ajman University, Ajman P.O. Box 346, United Arab Emirates; 9 King Fahd Medical Research Center, and Yousef Abdullatif Jameel Chair of Prophetic Medicine Application, Faculty of Medicine, King Abdulaziz University, Jeddah 21589, Saudi Arabia; 10 Biotechnology Division, National Centre for Disease Control, New Delhi 110054, India

**Keywords:** colorectal cancer, protein–protein interaction, sub-network, regulators, bottleneck-hubs, miRNAs, transcription factors

## Abstract

Colorectal cancer is one of the leading causes of cancer-associated mortality across the worldwide. One of the major challenges in colorectal cancer is the understanding of the regulatory mechanisms of biological molecules. In this study, we aimed to identify novel key molecules in colorectal cancer by using a computational systems biology approach. We constructed the colorectal protein–protein interaction network which followed hierarchical scale-free nature. We identified *TP53*, *CTNBB1*, *AKT1*, *EGFR*, *HRAS*, *JUN*, *RHOA*, and *EGF* as bottleneck-hubs. The *HRAS* showed the largest interacting strength with functional subnetworks, having strong correlation with protein phosphorylation, kinase activity, signal transduction, and apoptotic processes. Furthermore, we constructed the bottleneck-hubs’ regulatory networks with their transcriptional (transcription factor) and post-transcriptional (miRNAs) regulators, which exhibited the important key regulators. We observed miR-429, miR-622, and miR-133b and transcription factors (*EZH2*, *HDAC1*, *HDAC4*, *AR*, *NFKB1*, and *KLF4*) regulates four bottleneck-hubs (*TP53*, *JUN*, *AKT1* and *EGFR*) at the motif level. In future, biochemical investigation of the observed key regulators could provide further understanding about their role in the pathophysiology of colorectal cancer.

## 1. Introduction

Different types of cancers are the leading causes of death worldwide, and the mortality rate of colorectal cancer (CRC) is the second highest (9.4%) (https://gco.iarc.fr/today/explore) accessed on 1 March 2022. The rate of incidence and mortality of CRC is increasing rapidly with an estimate of 60% by the year 2030 [1]. The cumulative risk rate of death from CRC is 0.65% (in men) and 0.45% (in women). CRC, which results from the gradual accumulation of genetic and epigenetic changes leads to normal colonic mucosa transformation to adenocarcinoma [2]. CRC is caused by mutations in different genes (target oncogene, tumor suppressors, and genes involved in DNA repairing mechanism) which mainly affect the signaling pathways [3]. These related genes lead to uncontrolled cell cycle progression with the inactivation of the apoptosis process [3]. There are three molecular pathway disruptions that lead to CRC pathogenesis: (i) chromosomal instability (CIN), (ii) microsatellite instability (MSI), and (iii) CpG island methylation phenotype [4]. Along with these, types of CRC pathogenesis, mismatch repair (MMR), and translocation have been identified to affect important mechanisms/pathways (WNT, MAPK/PI3K, TP53, and TGF-beta). In addition, the presence of gene mutations (c-MYC, BRAF, KRAS, NRAS, PIK3CA, SMAD4, and P53) and alteration in non-coding RNAs like miRNAs (miRNA-31, miRNA-146, miRNA-147b, and miRNA-1288) can be used as therapeutic markers for patient outcome.

Various high-throughput genomics technologies have been extensively employed to study the involvement of genes and pathways associated with CRC pathogenesis [5]. In the development of drugs, computational methods have been widely utilized to understand disease mechanisms [6,7,8]. The systems biology approach has been used to identify gene network signatures for CRC (TP53, PCNA, and IL8 sub-network (SN)) related to apoptosis, DNA repair, and immune response, respectively [9]. Due to the involvement of multiple genes and pathways, the understanding of CRC pathogenesis is still unclear.

The protein–protein interaction (PPI) networks and gene-regulatory network topological studies provided an understanding of key molecules which control the overall integrity and functionality of neighboring proteins [10], SN [11], and related diseases [12,13]. Network theory has been proposed to play a significant role in understanding the complex regulatory network dynamics. From the different types of networks (scale-free, random, small-world, and hierarchical), the hierarchical network type gained special attention from biologists because hubs (high-degree nodes), SN (clusters of nodes), and self-organization are the important structural units of such networks [14,15]. Previous studies based on network theory approaches identified some central genes in CRC–protein–protein interaction (CRC–PPIN) using some topological parameters such as degree centrality, betweenness centrality, closeness centrality, and stress [16,17,18].

From a list of CRC-related genes, we constructed a CRC–PPI network to identify the key regulatory molecules (hubs, bottlenecks, and bottleneck-hubs), functionality of SN, transcriptional (transcription factors: TFs) and post-transcriptional (micro-RNA: miRNA). We also showed the crosstalk between bottleneck-hubs (Bn-Hs) and SN, which is another level of regulation that helps to maintain the CRC network stability. The PPIN and gene-regulatory network study helps us to understand the systematic regulation of the CRC gene, which followed a complex regulatory mechanism. We also predicted the key regulatory switches in between Bn-H-TFs-miRNAs which could influence the disease CRC.

## 2. Material and Methods

### 2.1. Network Statistical Analysis of Colorectal Cancer

The proteins associated with colorectal cancer were retrieved from the KEGG database [19]. The PPI of CRC proteins was retrieved from the STRING database [20]. The topological properties of the CRC–PPIN were characterized by using three topological parameters (degree distribution, clustering coefficient, neighborhood connectivity) and three centrality parameters (betweenness, closeness, eigenvector). We analyzed the constructed network by using Network Analyzer [21], a plugin in Cytoscape v3.8.2.

#### 2.1.1. Degree (*k*) and Probability of Degree Distribution (P(*k*))

Degree (*k*) is a basic characteristic that has an impact on a node’s centrality and is represented by the number of connections of a node to others in the network. The probability of degree distribution (*P*(*k*)) is represented by the given equation (Equation (1)).

(1) $$P\left(k\right)=\frac{N_{k}}{N}$$

where $N_{k}$ represents the total number of nodes with degree k and *N* represents a node.

#### 2.1.2. Clustering Coefficient *C*(*k*)

Clustering coefficient C(*k*) defines the strength of internal connections between the node’s neighborhood with the overall organization of the formation of clusters in the network. For a particular node, it is calculated using $C\left(k_{i}\right)=2m_{i}/k_{i}\left(k_{i}-1\right)$, where $m_{i}$ represents the total number of connections with its close neighbors.

#### 2.1.3. Neighborhood Connectivity $C_{N}\left(k\right)$

Neighborhood connectivity $C_{N}\left(k\right)$ is the number of neighbors connected with a node, and it defines the correlation pattern of connectivity for the interacting nodes of the network. $C_{N}\left(k\right)$ can be calculated using Equation (2).

(2) $$C_{N}\;\left(k\right)=\underset{q}{\sum }qP\;(q|k)$$

where P(q|k) is the conditional probability of creating a link from a node with a k degree to another node having a q degree [22].

#### 2.1.4. Betweenness Centrality $\left(C_{B}\left(v\right)\right)$

It measures a node occurring several times to bridge along the shortest path between nodes i to j. It is calculated using Equation (3).

(3) $$C_{B}\left(v\right)=\underset{i,j;i\neq j\neq k}{\sum }\frac{d_{ij}\left(\nu \right)}{d_{ij}}$$

where $d_{ij}\left(v\right)$ denotes the number of geodesic paths connecting node i to node j passing through node v. A high betweenness value indicates that the node lies on a path with many other nodes and has the significant ability to propagate information in the network [23].

#### 2.1.5. Closeness Centrality $\left(C_{c}\right)$

It is defined in terms of ‘shortest path lengths’ among the pair of nodes in a network. It can be calculated in terms of farness and is given using Equation (4).

(4) $$C_{C}\;\left(k\right)=\frac{N}{\sum_{j}d_{ij\;}}$$

where $d_{ij}$ is the geodesic distance between the pair of nodes *i* and *j*, and N is the nodes present in the network.

#### 2.1.6. Eigenvector Centrality $\left(C_{E}\right)$

It is proportional to the total sum of the centrality of all neighborhood nodes. It describes the effect of a node on signal processing. It is calculated using Equation (5).

(5) $$C_{E}\;\left(i\right)=\frac{1}{\lambda }\underset{j=nn\left(i\right)}{\sum }v_{j}\;$$

where *nn*(i) represents the nearest neighbor of the *i* node in the network, with eigenvalue λ and eigenvector $v_{i}$ of the eigenvalue equations, $Av_{i}=\lambda v_{i}$ where A is the network adjacency matrix.

### 2.2. Tracing of Bottleneck-Hubs

In a CRC–PPIN, the nodes with a high degree were considered as hubs [24,25,26,27] and high betweenness centrality (BC) value nodes as bottlenecks (Bn). Here, we filtered the nodes based on high degree and betweenness, which were called bottleneck-hubs (Bn-Hs). Bn-Hs play an important role in information flow and controlling capability in the network [28]. We selected the top 10 with the highest degree (k) and BC nodes from the constructed network. From the top 10 nodes in each category, we considered overlapped nodes as bottleneck-hubs for further analysis.

### 2.3. Detection of Subnetwork and Key Mediator Bottleneck-Hub

In our analysis, we used Molecular Complex Detection (MCODE) v2.0.0 [29] to identify the nodes that are highly interconnected in the form of SN. To separate the dense areas according to provided parameters, the approach uses vertex weighting by local neighborhood density and outward traversal from a locally dense seed protein. We used the default parameters of MCODE, a node score cutoff (0.2), haircut, node density cutoff (0.1), K-score (2), and maximum depth (100). The interaction between the Bn-Hs and the subnetworks was identified using Cytoscape v3.8.2. In this, study we focused on identifying the functional dependency between the Bn-Hs and SNs. Here, we calculated the interacting strength of each Bn-H with the highest scoring SN, which may provide the internal stability of PPIN. The Bn-H could be the most influencing node and become a vital means for communication between its interacting SNs, which may result in a more accurate understanding of the biological functions [30].

### 2.4. Functional Analysis of Subnetworks

We performed Gene Ontology (GO) for nodes forming the SNs using the g:Profiler package [31] to relate their biological significance. The g:Profiler tool executes the statistical enrichment analysis to predict the over-representation of information from GO terms such as molecular function (MF), biological processes (BP), cellular component (CC), PPI, biological pathways, and gene–disease association. In our study, we included GO terms (BP, MF, CC) for identified SN. We used the default parameters, such as the domain size set to “only annotated”, default g:SCS method (for multiple testing correction of p-values), p-value (0.05), and numeric IDs as prefix ENTERZGENE_ACC. We represented the GO enrichment analysis results of SNs in the form of a Manhattan plot.

### 2.5. Pathway Analysis of Bn-Hs

We conducted the GO enrichment analysis to expound the potential pathways of Bn-Hs involved in CRC. We performed the analysis of eight Bn-Hs (*TP53*, *CTNBB1*, *AKT1*, *EGFR*, *HRAS*, *JUN*, *RHOA*, and *EGF*) using the Enricher tool (https://maayanlab.cloud/Enrichr/#), accessed on 1 March 2023, containing 210 gene set libraries [32]. The results were sorted based on the p-value. We selected the top 10 highly enriched pathways and showed their odds ratio and combined score in the form of a table.

### 2.6. Construction of a Bn-H Regulatory Network

The combinatorial network of CRC included transcriptional and post-transcriptional regulatory molecules’ TFs and miRNAs, respectively. To identify the miRNA targets of Bn-Hs, we used two computational-based miRNA target prediction tools (TargetScan v8.0 and miRDB V6.0) [33,34] and one experimental validated database (miRTarbase) [35]. We considered the miRNA which was predicted by all three databases. Further, to identify the relation between miRNA-TFs, and TFs-Bn-H, we used the TransmiR v2.0 and TRRUST databases, respectively [36,37]. The regulatory function of TFs with Bn-H and miRNAs is categorized as (i) activators and (ii) repressors. Finally, we constructed the combinatorial regulatory network for Bn-H of colorectal cancer with TFs and miRNAs and visualized using Cytoscape v3.8.2 [38].

#### Coherent and Incoherent Feed-Forward Loops

Various types of topological motifs are found in large-scale biological networks, which are formed by a great variety of interactions between biological molecules. It is interesting to understand the dynamic behavior of CRC gene regulatory networks at the transcriptional and post-transcriptional level and to understand the importance of significant recurring wiring, known as the “network motif” [39,40,41]. The pattern of network motifs, such as the feed-forward loop associated with Bn-Hs, is termed as “coherent”, meaning the symbol of the regulation path (from TF to Bn-H) is similar to the overall sign of the indirect regulatory path (from TF through miRNA to the gene) [40,42]. If the sign of the directed and in-directed regulation were opposite, the network motif is considered as an incoherent type [40]. Both of these coherent and incoherent FFL motif behaviors were signed sensitive, and they selectively responded to stimulus steps in two ways—either more quickly or more slowly, depending on the sign [30,40].

## 3. Results

### 3.1. Hierarchal Scale-Free CRC-PPIN Topology

**Figure 1:**
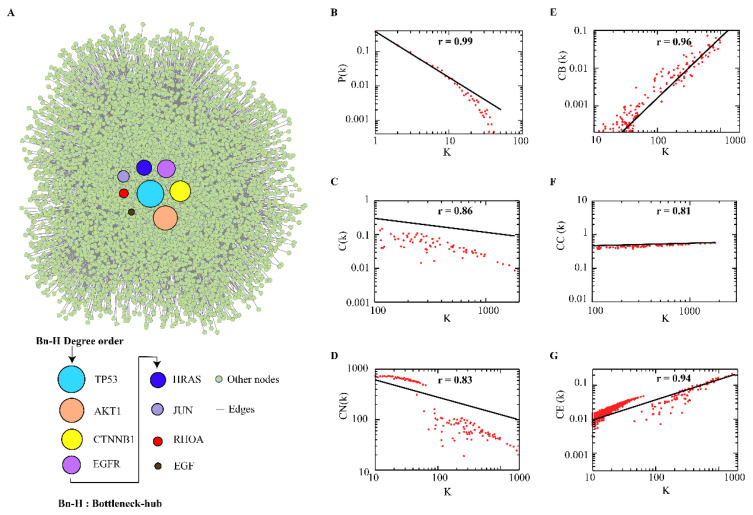
**Central proteins and behavior of CRC-PPIN**. (**A**) Overlapped nodes (8) in-between top 10 highest degree (k) and betweenness (C_B_) centrality parameters were considered as Bn-Hs and play an important role in information flow and controlling capability in the network. (**B**–**G**) All topological properties followed power law distribution and provided a scale-free topology (presence of lesser no. of nodes having larger degree).

We extracted 86 colorectal cancer-related proteins from the KEGG database which to further fetch the interacting partners in the STRING database. The constructed CRC–PPIN consists of 6556 nodes and 32,048 edges (Figure 1A). The statistical analysis of the topological parameters’ degree and BC of all 6556 nodes is given in (Supplementary Table S1). The statistical parameters of the CRC–PPIN followed power-scaling behavior against k. In overall analysis, (*P*(*k*)) obeys power law distribution $P\;\left(k\right)\sim \;k^{-\gamma }$ with a value of exponent γ=0.38 ±1.33 (Figure 1B), where the regression line fitted with the curve to the data point with $P\;\left(k\right)\sim \;k^{-1.33},$ with correlation coefficient (r) 0.92 fitted with the data. The value γ (1.33) provides hierarchical scale-free behavior to the network. The clustering coefficient C(*k*) also followed the power law scale as a function of degree $C\left(k\right)∼k^{-\alpha }$ with a negative exponent value of (α=2.09±0.41), which showed that the CRC–PPIN follows a hierarchical nature. The straight line fitted curve with $C\left(k\right)∼k^{-0.41}$ results from a coefficient value (r=0.86) correlated to the data set (Figure 1C). The neighbourhood connectivity $C_{N}\left(k\right)$ showed a negative exponent value (β =1355.22±0.34 ) given using the power-law fitting model $C_{N}\left(k\right)\sim k^{\beta }$. The fitted curve line with $C_{N}\left(k\right)\sim k^{-0.34}$ gives the correlation coefficient (r=0.83) (Figure 1D). The network showed a disassortative nature due to the calculated negative value of $\beta_{0}$, the exponent of connectivity parameter, and reflects that the Bn-Hs are still a significant part of regulating the stability of the network. To recognize the importance of the Bn-H nodes’ strength in signal processing in a network, we used three topological centrality parameters, such as closeness centrality $\left(C_{c}\right)$, betweenness centrality $\left(C_{B}\right)$ and eigenvector $\left(C_{E}\right)$. In the CRC–PPIN, these parameters followed power law against degree (K) and showed positive exponents values, indicating the strong regulating behavior of the leading Bn-H. The calculated values of exponents and correlation coefficients (r) are $C_{B}\left(\varepsilon =1.60,r=0.96\right)$, $C_{c}(\eta =0.76,r=0.81,\;C_{E}\left(\delta =0.59,\;r=0.94\right),\;\mathrm{respectively}$ (Figure 1E–G). The graph of betweenness against degree showed that high-connecting nodes have more controlling strength to outspread signals throughout the network. Our network followed a hierarchical scale-free nature and means that the network has a modular structure and system level of organization.

### 3.2. Central Bottleneck-Hubs

The Bn-Hs are the most influencing proteins and provide stability and control of the flow of information in the network. In the CRC–PPIN, we found the top 10 nodes as hubs (*TP53*, *AKT1*, *CTNNB1*, *EGFR*, *HRAS*, *JUN*, *MAPK3*, *RHOA*, *EGF*, and *KRAS*) and the top 10 nodes as bottlenecks (*TP53*, *CTNNB1*, *AKT1*, *EGFR*, *CYCS*, *RHOA*, *JUN*, *HRAS*, *EGF*, and *FOS*). Eight nodes (*TP53*, *AKTI*, *CTNNB1*, *EGFR*, *HRAS*, *JUN*, *RHOA*, and *EGF)* are common in both the parameters (K and BC) and were considered as Bn-Hs (Table 1). Node *TP53* showed the highest degree (1817) and BC (0.19876) in the CRC–PPIN. The Bn-H proteins function as the backbone of the network and have a great influence on information flow with more control over the network (Figure 1A). Mutations in such potential regulators (Bn-Hs), as well as deregulation of their expression, could alter protein interactions, influencing multiprotein complex formations and signaling pathways, disrupting system dynamics and resulting in tumor development in CRC and other associated diseases.

### 3.3. Subnetworks and Their Cross-Talk with Bottleneck-Hub

**Figure 2:**
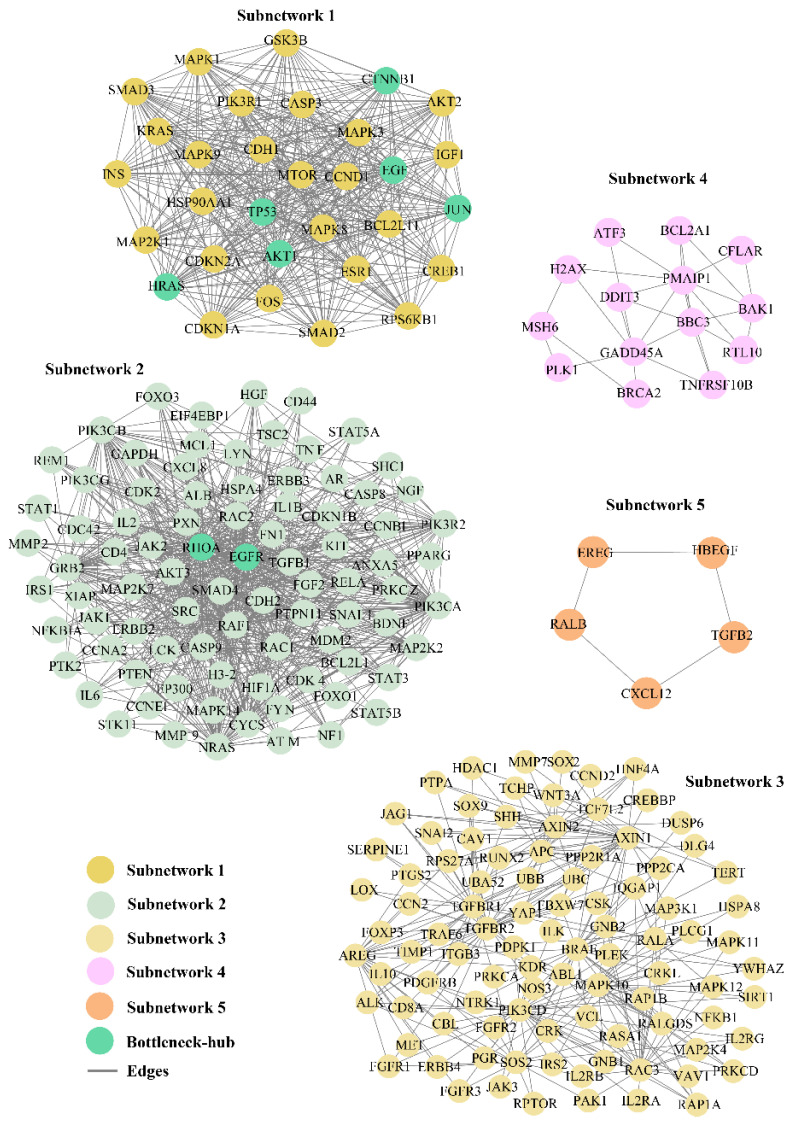
**Functionally significant highly interconnected regions.** In the CRC–PPIN, top five sub–network was selected on the basis of M–score. Eight Bn–Hs were also found in two subnetworks (1 and 2) which not only control the internal regulation of their own subnetworks, but also influence other subnetworks by interacting with different nodes.

**Figure 3:**
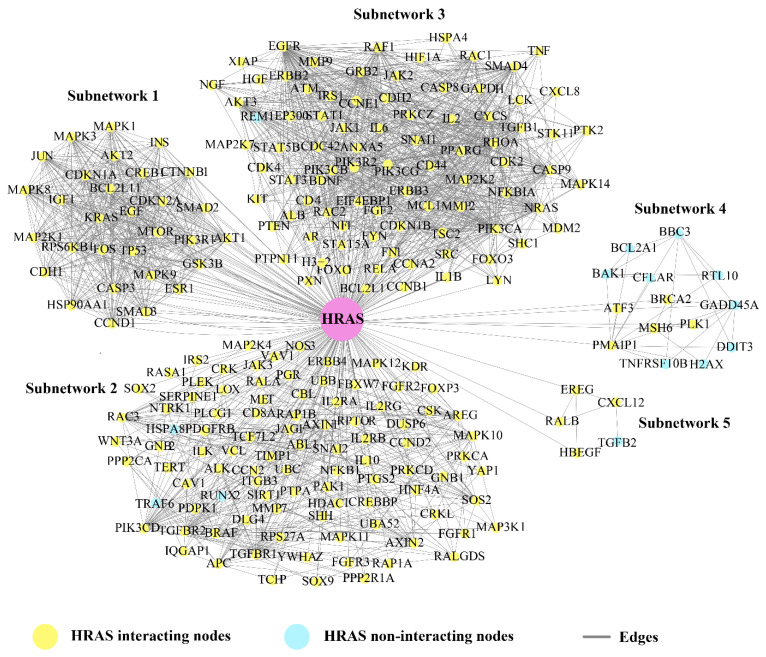
**Key mediator protein**. Bn–H (*HRAS*) acted as key mediator for subnetworks crosstalk and was helpful for signal processing in-between unconnected proteins. *HRAS* showed the highest in–teracting strength in between other Bn–Hs.

In the CRC–PPIN, we identified the five SNs that are highly interconnected clusters of nodes representing corresponding stable units that function as a single entity in the network (Figure 2). The SN-1 had 31 nodes and 422 edges with the highest M-scoring value (28.13). The second, third, fourth, and fifth SNs consisted of 86, 95, 14, and 5 nodes, respectively, with the corresponding edges of 681, 284, 28, and 5 (Figure 3). These SNs have a system-level organization that was maintained by connecting with other nodes and provided overall functionality to the network [43]. We found six (*TP53*, *AKT1*, *CTNBB1*, *HRAS*, *JUN*, and *EGF*) and two Bn-Hs (*EGFR* and *RHOA*) present in SN-1 and 2, respectively, which not only controls the internal regulation of their SN but also influences other subnetworks by interacting with different nodes. SNs 3, 4, and 5 showed the absence of Bn-Hs, suggesting that Bn-Hs are indirectly connected with the modular function of these three SNs (Figure 2). Crosstalk between the SNs may be possible due to the interaction with common Bn-Hs, and the removal of such regulators can affect the functionality of SNs and lead to the distortion of the network.

Based on the crosstalk between Bn-Hs and SNs, we found *HRAS* showed the highest strength of interaction (216) with all five SNs, followed by *TP53*, *EGFR*, *JUN*, *AKT1*, *CTNNB1*, *EGF*, and *RHOA* (Figure 3 and Table 2). Three Bn-Hs (*HRAS*, *TP53*, and *JUN*) showed more connections (30 nodes each) in SN-1, and also *HRAS*, *TP53*, and *JUN* showed the highest connection in SN-2 (85, 83, and 83), respectively, in comparison with other Bn-Hs. (Figure 3 and Table 2). Both *HRAS* and *TP53* also showed high control of the SN-3 and SN-4 with an interaction strength of 92 and 14, respectively. Surprisingly, *HRAS* also showed interactions with 5 Bn-Hs (*TP53*, *AKT1*, *CTNBB1*, *JUN*, and *EGF* in SN-1) and two Bn-Hs (*EGFR* and *RHOA* in SN-2) in the CRC–PPIN. The SN-5 is highly controlled by *EGFR*, *AKT1*, and *RHOA* with interaction strength (5).

SN-2, 3, and 4 are least controlled by *EGF* and *RHOA*, whereas in SN-5, RHOA has the highest number of interacting strengths (5) (Table 2). The Bn-Hs TP53 and *AKT1* showed high connections with SN-4 and 5, with an interaction strength of 14 and 5 nodes, respectively. In the overall result from the analysis of the Bn-H and SN interaction, we found *HRAS*, *JUN*, *TP53*, *EGFR*, and *AKT1* are the significant Bn-Hs present in the CRC–PPIN. We also found among all eight Bn-Hs that *HRAS* not only controls the other five Bn-Hs (*TP53*, *AKT1*, *CTNBB1*, *JUN*, *and EGF*), but that it is also performing their important role in SN-1.

### 3.4. Gene Ontology (GO) Analysis of Subnetworks

**Figure 4:**
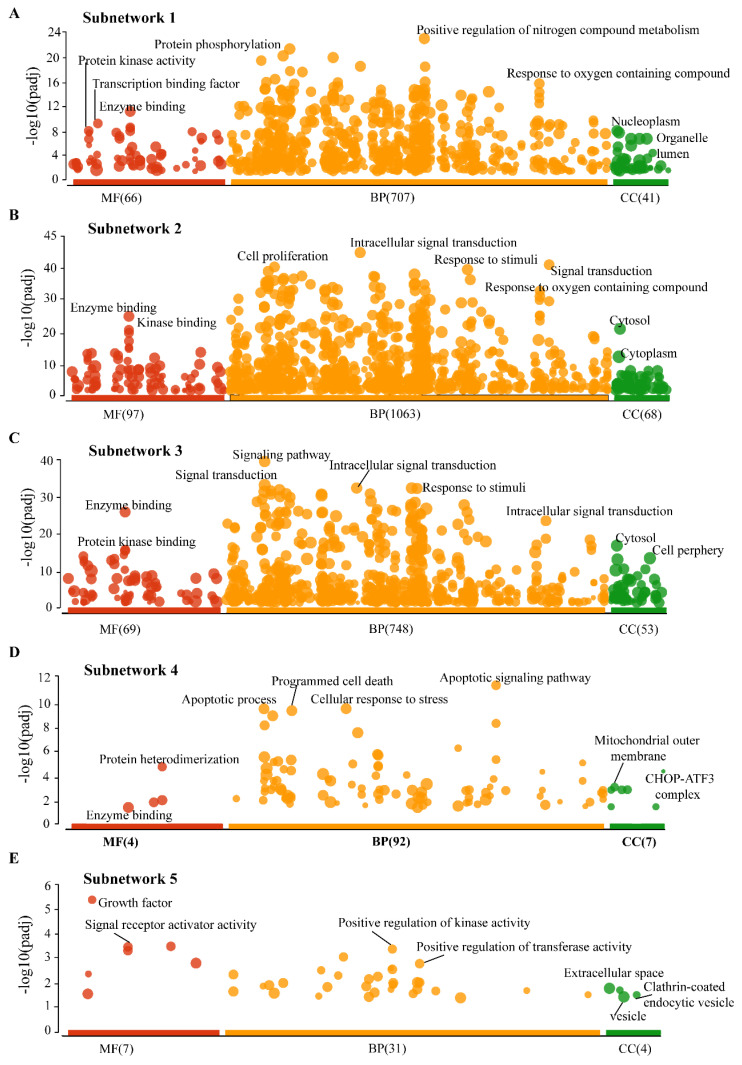
**Cellular role of Subnetworks.** (**A**) Functional enrichments showed that the MF of SN1 proteins associated with (phosphorylation, protein kinase activity, and transcription binding biological) processes, BP related to (positive regulation of nitrogen compound metabolism) processes in nucleoplasm and organelle lumen. (**B**) The MF of SN-2 were involved in enzyme and kinase binding-like activity, and in cytoplasm the BP of these protein enriched with cell proliferation, signal transduction, responses to stimuli, and intracellular signal transduction activity. (**C**) The proteins of SN-3 functionally enriched with enzyme and kinase binding-like activity, the BP mostly associated with signal transduction activity in cytosol and cell periphery. **(D**) The SN-4 proteins are located in the mitochondrial outer membrane and CHOP–ATF3 complex and involved majorly in apoptotic process and protein heterodimerization. **(E)** SN-5 were functionally enriched with extracellular space, the clathrin-coated endocytic vesicle to perform MF (growth factor, signal receptor activity), and BP such as the positive regulation of kinase and transferase activity. The sizes of the filled circle according to the term size—means larger terms have larger circles.

We performed the GO for all five SNs to understand their functional importance in the CRC–PPIN. SN-1 was functionally enriched with proteins that have protein phosphorylation, protein kinase activity, and transcription binding and that are related to biological processes such as the positive regulation of nitrogen compound metabolism and also a response to oxygen-containing compounds. SN-1 and associated proteins were found to be localized in the nucleoplasm and organelle lumen (Figure 4A). SN-2 and 3 were found to have similar functions, such as enzyme and kinase binding-like activity, and its proteins found to be biologically involved in cell proliferation, signal transduction, responses to stimuli, and intracellular signal transduction activity in the cytoplasm (Figure 4B,C). SN-4, the proteins’ molecular function enriched with protein hetero-dimerization, enzyme binding, and their biological processes were found to be like apoptotic processes and cellular stress responses. The SN-4 proteins are mainly located in the mitochondrial outer membrane and CHOP–ATF3 complex (Figure 4D). Associated proteins with SN-5 were mainly presented in extracellular space, the clathrin-coated endocytic vesicle to perform molecular functions (growth factor, signal receptor activity), and biological processes such as the positive regulation of kinase and transferase activity (Figure 4E). We also observed that 8 Bn-Hs presents in SN-1 and 2 were functionally enriched with protein phosphorylation, kinase activity, signal transduction, and apoptotic processes, suggesting their significance in their own SNs.

### 3.5. Highly Enriched Pathway Associated with Bn-Hs

From the pathway enrichment analysis, we identified that eight Bn-Hs (*TP53*, *AKTI*, *CTNNB1*, *EGFR*, *HRAS*, *JUN*, *RHOA*, and *EGF*) were mostly associated with signaling by ERBB2, non-receptor tyrosine kinases, and the ESR-mediated, extra-nuclear estrogen signaling pathways shown in (Table 3). Previous studies reported that gene mutations pinpointed in EGFR/MAPK, Notch, PI3K, TGF-β, and Wnt signaling pathways showed dysregulation in CRC [44]. So, understanding the relationships between these pathways and Bn-Hs may promote the development of new therapeutic or preventive CRC approaches.

### 3.6. Combinatorial Regulatory Network of Bottleneck-Hubs

**Figure 5:**
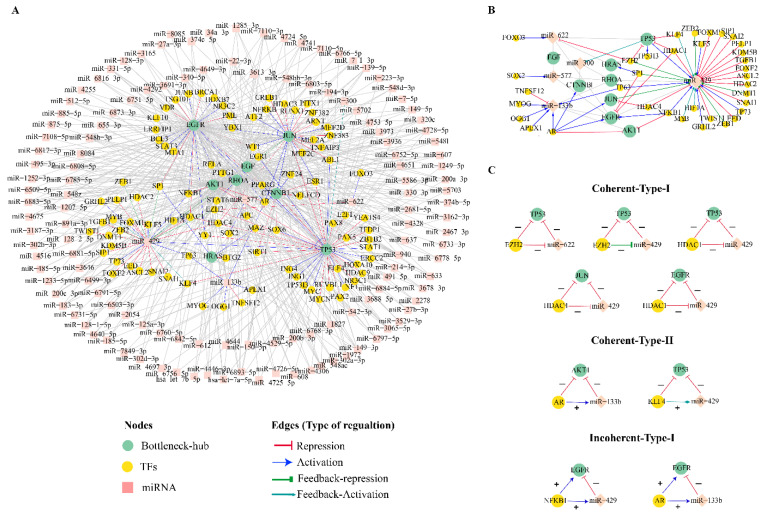
**Regulatory patterns of Bn-Hs in combinatorial network.** (**A**) The top 8 Bn-Hs transcriptionally and post-transcriptionally regulated by numbers of TFs and miRNAs. (**B**) Common miRNAs and TFs targeting Bn-Hs and regulating their function in terms of upregulation, downregulation, and feedback activation. Few TFs regulate the function of more than one Bn-H and their miRNAs, for e.g., *HDAC1* downregulating *TP53* and *EGFR* and miRNA-429. (**C**) Recurring gene circuits. Three node motif regulation categories incoherent type 1, coherent type 2, and incoherent type 3 FFLs.

The miRNA-target prediction tools, miRDB V6.0 and TargetScan v8.0, predicted 626 and 2239 miRNAs target Bn-Hs, respectively. The experimentally validated mirTarbase database showed that 393 miRNAs target Bn-Hs. All these three programs depicted 135 common miRNA target Bn-Hs, which were further used to identify TFs using the TransmiR database. Out of these 135 miRNAs, seven miRNAs (miR-6893-5p, miR-940, and miR-6808-5p, miR-6785-5p, miR-6883-5p, miR-149-3p and miR-4728-5p) target six Bn-Hs (*AKT1*, *EGF*, *EGFR*, *JUN*, *RHOA*, and *TP53* (Figure 5A).

We found only 36 TFs regulate 5 miRNAs (miR-622, miR-300, miR-577, miR-133b, and miR-429) out of 135 miRNAs using the TransmiR database. The miR-429 is highly regulated by the large number of TFs. We also retrieved 68 TF targets for 8 Bn-Hs and observed miR-429 is also post-transcriptionally regulated by two Bn-Hs, *TP53* and *JUN* (Figure 5B). The Bn-H regulatory network represented the relation between the Bn-Hs, TFs, and miRNAs shown in Figure 5A. The constructed Bn-Hs regulatory network consisted of 239 nodes and 596 edges (Figure 5A). From the analysis of the regulatory network, we extracted the regulatory relation between TFs and miRNA which were commonly regulating Bn-Hs. A few TFs (*EZH2*, *HDAC1*, *AR*) were found to regulate more than one Bn-H. Among all the Bn-Hs, *TP53* has the largest number of regulating targets (miRNAs:77 and TFs:37). The TFs (*EZH2*, *KLF4*, and *HDAC1)* directly repressed the transcription process of *TP53* (Figure 5B,C). Transcription factors *AR* and *NFKB1* both activated the *EGFR*, but *AR* also repressed the transcription process of *AKT1*. Furthermore, Bn-H and *JUN* were directly inhibited by *HDAC4* in the process of transcription (Figure 5B,C).

By the analogy of Bn-H, *TP53* and *JUN* also function as TF-targeting miR-429. *TP53* activates miR-429, whereas *JUN* showed a feedback repression mechanism against miR-429. *TP53*, post-transcriptionally was repressed by two miRNAs (miR-622 and miR-429) and *EGFR* was commonly repressed by miR-133b and miR-429. The miRNA miR-133b also represses the transcription process of *AKT1* (Figure 5B). The miRNA (miR-429) transcription is regulated by the feedback activation process by TF (*KLF4*).

### 3.7. Coherent and Incoherent Type Feed-forward Loops in the CRC Bn-H Regulatory Network

The Bn-H regulatory network of CRC consisted of a few network motifs as coherent (type I and II) and in-oherent types of the feed-forward loop (FFL) in-between TF:miRNAs and Bn-Hs. Incoherent type-I, the resultant regulation of the indirect path from TF to Bn-H (*TP53*, *JUN*, *EGFR*), was observed as a negative inhibition (EZH2:miR-622:*TP53*, *EZH2*:miR-429:*TP53*, *HDAC1*:miR-429:*TP53*, *HDAC4*:miR-429:*JUN*, and *HDAC1*:miR-429:*EGFR*) (Figure 5C). In the case of coherent type-II FFL, both *TP53* and *AKT1* were positively inhibited in indirect regulation by TFs (*AR*:miR-133b: *AKT1*, and *KLF4*:miR-429:*TP53*). In FFL incoherent type-I, TFs *NFKB1* and *AR* both activated the miRNAs (miR-429 and miR-133b), respectively, but these two miRNAs further repressed the Bn-H EGFR, so the net result of the effect of the indirect path from TF to gene is repression (Figure 5C).

## 4. Discussion

The term “targeted cancer therapies” refers to a new class of anti-cancer medications developed to block particular molecular targets thought to be essential for tumor development or progression [45]. The efficacy of targeted cancer therapies depends on the molecules selected as drug targets. It is important to provide a new therapeutic target for different types of cancers in order to have a much greater understanding of the mechanisms of the disease that contribute to the cell death that leads to CRC. To study these processes, researchers are modeling other elements of CRC, in a range of model organisms, directed by recent insights into its genetic and molecular basis. Animal models or cell cultures that are indicative of a carcinogenic condition in humans may be employed in studies related to the growth, treatment, and prevention of CRC malignancies. There are mainly three molecular pathway disruptions that lead to CRC pathogenesis: chromosomal instability (CIN), microsatellite instability (MSI) of the CpG island methylation phenotype, and mismatch repair (MMR); translocation has been identified to affect important mechanisms/pathways (WNT, MAPK/PI3K, TP53, and TGF-beta). Animal models are important for studies of the development and pathogenesis of colorectal tumors, as well as for the evaluation of possible risk factors, preventive agents, and treatments. Current studies revealed that chemotherapy and anti-epidermal growth factor receptor therapy are recommended for microsatellite stability or proficient mismatch repair left-sided treatment [46].

Here, we applied a computational systems biology approach which is less-time consuming, more cost effective, and coincides with the growing demand for developing targeted therapeutics. It could be used to predict the CRC target regulators and their robustness in maintaining self-organized behavior, as well as unravel the challenges of signaling involved in the basic processes of cellular death, survival, and developing strategies to stimulate cancer cells.

We constructed and analyzed the CRC–PPIN and identified the Bn-Hs. Further, the regulatory network (TF and miRNA) targets of the identified Bn-Hs were constructed. The statistical results of the CRC–PPIN revealed the hierarchical scale-free nature of the network. Overlapped nodes in between the top 10 highest K and BC were considered as Bn-Hs (*TP53*, *CTNBB1*, *AKT1*, *EGFR*, *HRAS*, *JUN*, *RHOA*, and *EGF)* in the CRC–PPIN (Figure 1A). The Bn-Hs are the most influencing proteins and provide stability and control of the flow of information in the network. The network showed a disassortative nature due to the calculated negative value of $\beta_{0}$; the exponent means that the Bn-H is still a significant part of regulating the stability of the network. Based on Bn-H and SN interactions, we found among the Bn-Hs that *HRAS* showed the highest strength of interaction (216 edges) with all five SNs, indicating it as the key mediator of the subnetworks (Figure 3). In SN-1, functionally related to protein phosphorylation, protein kinase activity and transcription binding, *HRAS* controls the function of SN-1 with five other Bn-Hs (*TP53*, *AKT1*, *CTNBB1*, *JUN*, and *EGF*). The relationship between Bn-H and SN interactions was involved in the regulation of the CRC–PPIN. The network’s crosstalk for these key proteins (Bn-Hs) as well as functional SNs is likely an attempt to maintain structural aspects of the network that facilitate disease biology.

The eight Bn-Hs (*TP53*, *AKTI*, *CTNNB1*, *EGFR*, *HRAS*, *JUN*, *RHOA*, and *EGF*) identified using computations were already known for the CRC and validated by many previously studied experimental evidence. To check their association with other types of cancers, we used the versatile platform “disgenet2r”, an R package, and the literature [47]. We found Bn-Hs associated with CRC were also related to other types of cancers such as liver carcinoma, osteosarcoma, breast cancer, pancreatic cancer, lung cancer, ovarian neoplasm, brain neoplasms, and oesophageal neoplasm [47,48,49]. In CRC, the *TP53* gene is mutated in 43% of tumors, and the remaining tumors often have compromised p53 functioning due to changes in regulatory mechanisms [50]. The gain of function and loss of function activities of mutated *TP53* is linked to cell proliferation, metastasis, and invasion that is further associated with CRC progression and other types of cancers [50,51,52]. Interactions of p53 with other transcription factors can enhance or repress their activity. Interaction of mutant p53 with the STAT3 was associated with STAT3 phosphorylation, JAK2/STAT3 signaling process, and CRC cells proliferation [53]. Additionally, mutant p53 also interacts with another transcription SP1; this PPI has been shown to regulate cell migration, metastasis, angiogenesis, and chemoresistance [54]. p53 knockout animals with tumor-inducer AOM was efficient in inducing carcinogenesis in the colon of the animals [55,56]. One of the major causes of CRC pathogenesis is the activation and deregulation of the AKT/mTOR signaling pathway. Dysregulation in the *AKT1* gene, such as mutations, altered the function and/or its protein expression, thus modifying the response and sensitivity to cancers. The mutations altered the function of *AKT1* observed in various forms of human malignancies, such as breast, lung, bladder [57], and digestive tract malignancies [58,59,60]. *AKT1* protein plays an important role in the development of cancer therapies. *CTNNB1* is altered in 3.10% of all cancers, with lung, colon, prostate, and hepatocellular carcinomas having the highest rate of prevalence [61]. The majority of catenin mutations in colorectal cancer are homozygous [62]. *CTNNB1* functions as a coactivator downstream of the oncogenic Wnt signaling pathway, and mutations in this gene have been associated with oncogenesis in CRC [63,64,65]. *HRAS* is a GTP-binding protein that plays an important role in many cellular networks that control a variety of signaling pathways, such as growth regulation, proliferation, survival, differentiation, adhesion, and cell survival, all of which lead to many types of cancers on their disruption [66]. *HRAS* protein is majorly involved in the MAP-kinase signaling pathway [67]. *HRAS* is altered in 0.94% of all cancers such as bladder urothelial carcinoma, breast, lung, prostate, and colon adenocarcinoma [61]. K-ras^v12^ mutation alone is not capable of inducing tumorigenesis, but once it is associated with mutations in repair genes, such as the MSH2 gene, it promotes and accelerates tumor development [68,69]. *JUN* is a proto-oncogene also known as p39 involved in the regulation of gene expression. The *JUN* family gene c-Jun and its increased expression have been reported in human colorectal tumors [70]. In many cell types, c-Jun is found to function as a proliferation-promoting gene, and its activation is required for cell cycle progression and neoplastic transformation [71,72]. *JUN* is altered in 0.87% of all cancers due to missense, nonsense mutations, silent and frameshift insertions, and deletions are observed in cancers such as intestinal cancer, lung cancer, and skin cancer [61]. Downregulation of AP-1 gene expression is an initial event in the oridonin-mediated inhibition of colorectal cancer, as shown in studies in vitro and in vivo [73]. *RHOA* is a member of the small GTPase family of proteins that regulates a cell signaling pathway that connects plasma membrane receptors to the formation of focal adhesions and actin stress fibers. It has been linked to a variety of critical cancer-related processes in mammalian cells, including proliferation, migration, and survival [74]. *RHOA* expression in tumor samples is higher than in normal tissues [75]. *EGFR* is a gene that encodes for the epidermal growth factor receptor protein. EGFR is mainly associated with receptor tyrosine kinase/growth factor signaling [76]. Activating *EGFR* mutations enhances *EGFR* kinase activity, resulting in increased activation of downstream pro-survival signaling pathways [77]. It is commonly mutated and/or overexpressed in various types of human cancers and is the center of many cancer therapies currently used in clinical practice [78]. The higher degree nodes of the CRC PPI network corresponded to the majority of the Bn-Hs and essential genes, indicating their important role in the maintenance of the system and ultimately the survival of CRC cells. Mutations in two or more genes are often associated with tumor malignancy [79]. Loss of such important Bn-H from the CRC network may change the interaction pattern of proteins and can lead to the disintegration of the functional/pathway modules rendering the CRC cells weaker.

Regulatory network and understanding its topological properties help in clarifying the functional role of target-associated transcription factors and miRNAs, which could provide novel therapeutic targets, e.g., gene-regulated analysis based on gene expression data that revealed potential candidate genes for squamous lung cancer [80]. Various bioinformatic integrative analyses identified candidate target genes, miRNA, and TF as signatures in prostate cancer [28]. We integrated multi-omics analysis to evaluate transcriptome patterns to decode the system-level molecular signatures of the protein (Bn-Hs, TFs) and RNA levels (miRNAs). The results suggested that Bn-H was positively and negatively activated or repressed by a number of common TFs and miRNAs at the transcriptional and post-transcriptional level, respectively. The coherent and incoherent types of FFLs indicated the key targets (*TP53*, *JUN*, *EGFR*, and *AKT1*) and their mode of regulation by TFs and miRNAs. In the analysis of three node motifs, miR-429 was critical for CRC regulation and post-transcriptionally regulated Bn-Hs (*TP53* and *EGFR*, and *JUN*), and it is largely targeted by a number of TFs. *KLF4* transcription factor directly represses the *p53* transcription element in human breast cancer cells and lead to *p53* apoptosis [81]. Overexpression of *SIRT1* promotes HG-attenuated corneal epithelial wound healing via p53 regulation [82]. The repression of the transcriptional activity of the *AR* by *PTEN* is likely to involve the downregulation of *AKT1* [83]. *HDAC4* individually upregulate *JUN* promoter activity [84]. *AR* is the transcription factor known to be involved in CRC and it is regulating the transcription process as activator for three Bn-Hs (*AKT1*, *CTNNB1*, and *JUN*) [83,85,86,87]. The connection between the Bn-H, TFs, and miRNAs was found to be important for regulation, which controlled the overall network topology.

## 5. Conclusions

A computational systems biology approach could be used to predict CRC target regulators (Gene/TF/miRNA) and their robustness in maintaining self-organized behavior, as well as to unravel the challenges of signaling involved in the basic processes of cellular death, survival and to develop strategies to stimulate cancer cells. Network biology approaches such as the feed-forward loop (FFL) are effective for investigating the underlying global topological structures of molecular networks. We observed miR-429, miR-622, and miR-133b, and that transcription factors (*EZH2*, *HDAC1*, *HDAC4*, *AR*, *NFKB1*, and *KLF4*) regulate four bottleneck-hubs (*TP53*, *JUN*, *AKT1*, and *EGFR*) at the motif level. Our results indicated some insightful data as well as a few miRNA and TF candidates, as well as their regulation, for future experimental validation in CRC. The CRC-specific gene-miRNA-TF regulatory network will help to understand the complicated CRC regulatory processes and guide clinical treatment.

## Figures and Tables

**Table 1:** Topological properties of nodes presented in the CRC–PPIN.

S.NO	Name	Degree (K)	Name	Betweeness (C_B_)
1	TP53 *	1817	TP53	0.198765
2	AKT1 *	1623	CTNNB1	0.11618
3	CTNNB1 *	1426	AKT1	0.11499
4	EGFR *	1276	EGFR	0.096395
5	HRAS *	980	CYCS	0.072343
6	JUN *	966	RHOA	0.063284
7	MAPK3	908	JUN	0.054568
8	RHOA *	838	HRAS	0.0481
9	EGF *	814	EGF	0.047273
10	KRAS	801	FOS	0.041709

This table shows the top 10 highest degree nodes (hubs) and betweenness nodes (bottlenecks) out of the total (6556) nodes in the CRC–PPIN. The “*” highlighted the nodes considered as bottleneck-hubs used to identify the key mediator of the subnetworks’ crosstalk, which may be important for signal processing from the center to the periphery of the CRC–PPIN.

**Table 2:** The bottleneck-hub and their interaction strength with subnetworks in the CRC–PPIN.

Name of Bn-H	SN-1	SN-2	SN-3	SN-4	SN-5	Total
HRAS*	30	85	92	5	4	216
TP53	30	83	72	14	3	202
EGFR	29	80	77	7	5	198
JUN	30	83	70	10	3	196
AKT1	27	78	70	12	5	192
CTNNB1	27	77	73	6	3	186
EGF	29	75	59	1	4	168
RHOA	30	68	63	1	5	167

The relationship between Bn-H and SN interactions were involved in the regulation of the CRC–PPIN. HRAS* showed highest strength of interaction (216 edges) with all five SNs, indicating it as the key mediator of the subnetworks. Abbreviation: subnetwork (SN), bottleneck-hub (Bn–H).

**Table 3:** List of top 10 pathways associated with bottleneck-hubs.

S.no	Term	*p*-Value	Adjusted *p*-Value	Odds Ratio	Combined Score	Genes
1	Signaling by ERBB2 R-HSA-1227986	3.98 × 10^12^	9.44 × 10^10^	755.6061	19,833.86	EGF, AKT1, HRAS, EGFR, RHOA
2	Signaling by Non-Receptor Tyrosine Kinases R-HSA-9006927	5.43 × 10^12^	9.44 × 10^10^	707.2695	18,346.34	EGF, AKT1, HRAS, EGFR, RHOA
3	ESR-mediated signaling R-HSA-8939211	3.81 × 10^9^	4.42 × 10^7^	180.4098	3497.451	JUN, EGF, AKT1, HRAS, EGFR
4	Signaling by NOTCH R-HSA-157118	5.60 × 10^9^	4.87 × 10^7^	166.6162	3165.742	JUN, EGF, AKT1, TP53, EGFR
5	GRB2 events in EGFR signaling R-HSA-179812	9.22 × 10^12^	5.62 × 10^7^	1332.2	24,647.6	EGF, HRAS, EGFR
6	Extra-nuclear estrogen signaling R-HSA-9009391	1.13 × 10^8^	5.62 × 10^7^	288.7391	5283.369	EGF, AKT1, HRAS, EGFR
7	SHC1 events in EGFR signaling R-HSA-180336	1.20 × 10^8^	5.62 × 10^7^	1198.92	21,867.39	EGF, HRAS, EGFR
8	Constitutive signaling by EGFRvIII R-HSA-5637810	1.53 × 10^8^	5.62 × 10^7^	1089.873	19,615.82	EGF, HRAS, EGFR
9	GRB2 events in ERBB2 signaling R-HSA-1963640	1.91 × 10^8^	5.62 × 10^7^	999	17,757.53	EGF, HRAS, EGFR
10	Signaling by ERBB2 ECD mutants R-HSA-9665348	1.91 × 10^8^	5.62 × 10^7^	999	17,757.53	EGF, HRAS, EGFR

## Data Availability

All the data is available with the authors and shall be provided upon request.

## Supplementary Materials

The following supporting information can be downloaded at: https://www.mdpi.com/article/10.3390/ijms24065356/s1.

## Abbreviations

AR (Androgen receptor), AKT1 (AKT serine/threonine kinase 1), Bn-H (Bottleneck-hubs), BP (Biological Process), CC (Cellular component), CRC (Colorectal cancer), CRC-PPIN (Colorectal cancer- protein-protein interaction, CTNBB1 (Catenin beta-1), EGF (Epidermal growth factor), EGFR (Epidermal growth factor receptor), EZH2 (Enhancer of zeste homolog 2), HDAC1 (Histone deacetylase 1), HDAC4 (Histone deacetylase 1), HRAS (Harvey Rat sarcoma virus), JUN (transcription factor Jun), KLF4 (KLF transcription factor 5), KRAS (proto-oncogene), MF (Molecular function), miRNA (micro-RNA), NFKB1(Nuclear factor kappa B subunit 1), PPI (Protein-Protein Interaction), PPIN (Protein-Protein Interaction Network), RHOA (Ras homolog family member A), SN (Sub-network), TF (Transcription factor), TP53 (Tumor protein P53).
